# Hypoxia Compromises the Differentiation of Human Osteosarcoma Cells to CAR-R, a Hydroxylated Derivative of Lithocholic Acid and Potent Agonist of the Vitamin D Receptor

**DOI:** 10.3390/ijms26010365

**Published:** 2025-01-03

**Authors:** Haley Evans, Alexander Greenhough, Laura Perry, Gonzalo Lasanta, Carmen M. Gonzalez, Antonio Mourino, Jason P. Mansell

**Affiliations:** 1 School of Applied Sciences, College of Health, Science and Society, University of the West of England, Coldharbour Lane, Bristol BS16 1QY, UK; 2 Ignacio Ribas Research Laboratory, Department of Organic Chemistry, University of Santiago de Compostela, 15782 Santiago de Compostela, Spain

**Keywords:** osteosarcoma, lithocholic acid, vitamin D receptor, hypoxia, differentiation, alkaline phosphatase

## Abstract

The active metabolite of vitamin D3, calcitriol (1,25D), is widely recognised for its direct anti-proliferative and pro-differentiation effects. However, 1,25D is calcaemic, which restricts its clinical use for cancer treatment. Non-calcaemic agonists of the vitamin D receptor (VDR) could be better candidates for cancer treatment. In this study, we examined the influence of the hydroxylated lithocholic acid derivative CAR-R on osteosarcoma (OS) cell (MG63) growth and differentiation. Treatment of MG63 cells with CAR-R inhibited growth under conventional and hypoxic conditions. Co-treating cells with CAR-R and a lysophosphatidic acid (LPA) analogue resulted in their differentiation, as supported by synergistic increases in alkaline phosphatase (ALP) activity. Under hypoxic conditions, however, this differentiation response was attenuated. The importance of observed increases in hypoxia inducible factors (HIFs) were investigated through targeted disruption using pharmacological and genetic approaches. Disruption elicited a reduction in ALP activity, suggesting an important role for HIFs in OS differentiation. Finally, we examined the expression of the VDR protein. Hypoxic MG63s expressed less VDR, with the levels increasing with CAR-R exposure. Whilst these findings are encouraging, future studies aimed at bolstering the pro-differentiating effect of CAR-R under hypoxic conditions are warranted if this agent is to gain traction in the treatment of OS.

## 1. Introduction

**Figure 1:**
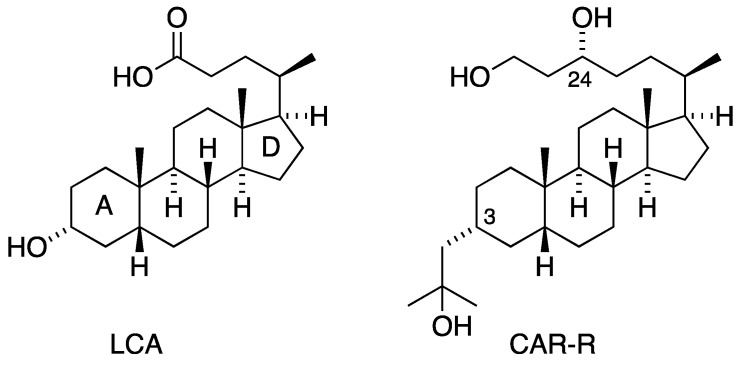
Structures of lithocholic acid (LCA) and the hydroxylated derivative, CAR-R.

Calcitriol (1,25D), the active metabolite of vitamin D, is widely recognised as an antiproliferative, pro-differentiating secosteroid hormone [1,2]. Upon entering the cytoplasm, 1,25D binds to the vitamin D receptor (VDR), the complex of which combines with the retinoid X receptor (RXR) to form a trimeric transcriptional composite. This complex translocates to the nucleus to influence the expression of target genes bearing vitamin D response elements (VDREs). Despite the clinical potential of 1,25D for the treatment of cancer [3,4], prolonged exposure will lead to hypercalcaemia, a debilitating condition that can be fatal. The calcaemic property of 1,25D has therefore stimulated the development of less-calcaemic surrogates including secosteroid compounds, such as EB1089 [5] and references therein], and derivatives of lithocholic acid (LCA), a secondary bile acid and agonist of the VDR [6]. With regard to the latter, there has been the recent development of side chain hydroxylated LCA derivatives, e.g., CAR-R (Figure 1), which displays a high affinity for the VDR [7]. The 3-hydroxyethyl group of CAR-R interacts with C-terminal VDR pocket residues, Tyr427 and Val444, resulting in a stable, active VDR conformation. Additional structural data with respect to the 3-hydroxylalkyl and dihydroxylated side chains indicated that CAR-R would be a potent VDR agonist.

Corroborating evidence was supported by anti-inflammatory in vitro studies. The inhibition of inflammation was assessed using primary human keratinocytes and quantification of the pro-inflammatory cytokine, CCL2. The exposure of CAR-R reduced the CCL2 secretion from cells stimulated with IL-4, IL-13, IL-22, and IFN-γ. The near-complete (96%) inhibition of CCL2 secretion was achieved with an IC50 of 0.15 nM [7]. Given these encouraging findings, this novel LCA derivative could form part of the arsenal in the treatment of cancers where expression of the VDR is evident, e.g., osteosarcoma [8].

Osteosarcoma (OS) is the most common primary bone cancer with an overall annual incidence rate of 3.1 per million. The 5-year survival rate continues to be around 20%, an outcome matching that reported back in the 1950s. Those most commonly affected are adolescents with a peak incidence of 4.2 per million, which then declines to 1.7 per million between the ages of 25–59 [9]. OS is often characterised by an undifferentiated osteoblastic phenotype associated with aggressive cancer cell behaviour and resistance to conventional therapies. Therefore, a potential approach to treating OS would be to induce the cancer cells to differentiate towards a more benign or normal state. There is emerging and compelling evidence that human OS could be managed by 1,25D; treating OS cells with 1,25D has been found to reduce proliferation, migration, and epithelial-to-mesenchymal transition [10]. However, given that 1,25D exerts a calcaemic action, potent, less calcaemic VDR agonists, like CAR-R, could be better candidates for treating OS.

In considering the application of CAR-R for OS cell differentiation, it is important to note that solid tumours, including OS, often experience hypoxia (low oxygen concentration) due to rapid growth and poor vascularisation. As with other cancers, hypoxia is known to fuel OS development and malignant progression, events driven by hypoxia inducible factors (HIFs) [11], a set of proteins which control the expression of genes linked to cancer cell fate [12], including the regulation of differentiation. It is therefore important to assess the potential of LCA derivatives on OS differentiation in the context of hypoxia. Of further significance are the multitude of studies demonstrating that the beneficial, antitumorigenic effects of 1,25D could be neutralised in other solid tumour types experiencing hypoxia [13]. Whether this is the case for OS has not been reported. Herein, we examined the ability of CAR-R to promote the differentiation of human OS cells (MG63) and to ascertain the influence of hypoxia in that process.

## 2. Results

### 2.1. CAR-R Displays Antiproliferative Activity

**Figure 2:**
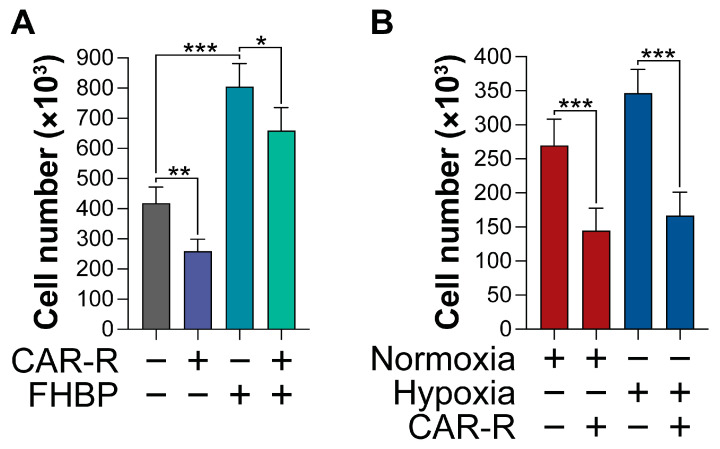
CAR-R exhibits antiproliferative activity. (**A**). MG63 cells were maintained under conventional culturing conditions for 72 h using a phenol red-free, serum-free medium supplemented with CAR-R (100 nM), FHBP (500 nM), or their combination. Cell number was determined biochemically using the MTS-PMS assay. As anticipated, cells stimulated with FHBP grew significantly in number compared to controls (*** *p* < 0.0001). In contrast, the treatment of MG63 cells with CAR-R led to a significant reduction in cell number (** *p* < 0.0001). CAR-R also attenuated the growth of cells to FHBP stimulation (* *p* < 0.01). The data are the mean plus the standard deviation from eight pooled, independent experiments (n = 32 for all groups). (**B**). Both normoxic and hypoxic cells respond to CAR-R (100 nM), exhibiting significantly fewer cells (*** *p* < 0.0001) compared to their corresponding controls. All data are expressed as the mean and standard deviation from seven, pooled, independent experiments (n = 36).

The exposure of MG63 cells to CAR-R (100 nM) resulted in significantly fewer cells (*p* < 0.0001) compared to the control cultures (Figure 2A). CAR-R, under conventional culturing conditions, was also able to modestly, yet significantly (*p* < 0.01) inhibit the pro-mitogenic effect of FHBP. CAR-R was also found to exert an antiproliferative effect on MG63s maintained under hypoxic conditions for three days (Figure 2B). The reduction in cell number for the different conditions was similar at approximately 47 and 52% for normoxic and hypoxic conditions, respectively.

### 2.2. Both DMOG and Hypoxia Compromise MG63 Differentiation

**Figure 3:**
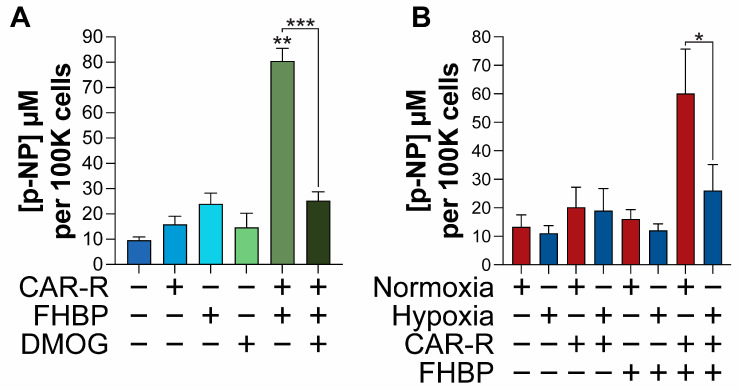
The pro-differentiating effect of CAR-R is inhibited by DMOG and hypoxia. (**A**). MG63 cells were grown under conventional culturing conditions for 72 h using phenol red-free, serum-free medium supplemented with either CAR-R (100 nM), FHBP (500 nM), or DMOG (250 micromolar). Cells were also co-treated with FHBP and CAR-R in the absence and presence of DMOG. Evidence of differentiation was via total alkaline phosphatase (ALP) activity with quantification of p-nitrophenol (p-NP) from p-nitrophenyl phosphate. The co-treatment of MG63 cells led to a clear, synergistic increase in p-NP compared to all other groups (** *p* < 0.001). When co-stimulated cells were also exposed to DMOG, there was a stark reduction in ALP activity (*** *p* < 0.001). The data are the mean plus the standard deviation from three pooled, independent experiments (n = 12 for all groups). (**B**). Exposure of cells to a hypoxic environment resulted in a similar outcome, with co-treated cells exhibiting significantly less ALP activity compared to normoxic cells (* *p* < 0.001). The data are the mean plus the standard deviation from three pooled, independent experiments (n = 12 for all groups).

Co-stimulation of MG63 cells with CAR-R and FHBP led to a clear and synergistic increase in ALP activity, a marker of cellular differentiation (Figure 3A). In contrast, co-stimulated cells treated with dimethyloxalylglycine (DMOG) displayed markedly reduced ALP activity (*p* < 0.001). Cells maintained under hypoxic conditions behaved in a similar manner, exhibiting significantly reduced (*p* < 0.001) ALP activity compared to normoxic cells (Figure 3B).

### 2.3. Hypoxia Upregulates the Expression of HIFs

**Figure 4:**
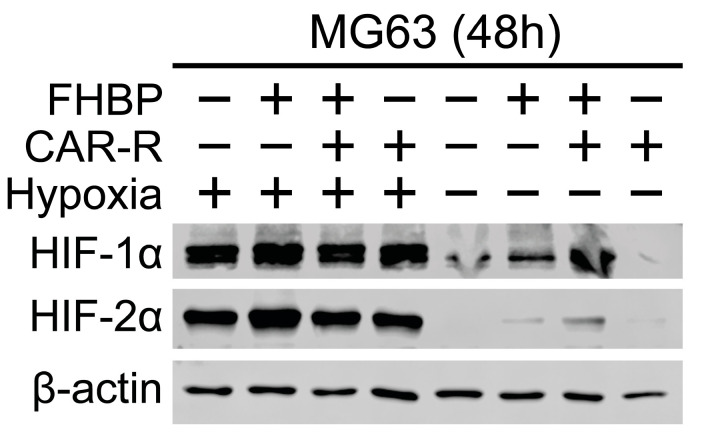
Hypoxia up-regulates the expression of hypoxia inducible factors (HIFs). MG63 cells, seeded into T25 flasks, were treated with either CAR-R (100 nM), FHBP (500 nM), or their combination under normoxic and hypoxic conditions for 48 h. Flasks were subsequently removed, the media aspirated, and the cell monolayers lysed on ice. Lysates were transferred to microcentrifuge tubes and processed for SDS-PAGE and Western blotting. Blots were probed for both HIF-1 and 2α alongside β-actin, which served to confirm equal sample loading.

MG63 cells maintained under hypoxic conditions for 2 days displayed clear increases in both HIF-1 and 2α (Figure 4). CAR-R-FHBP co-treated cells, under conventional, normoxic conditions, exhibited evidence of raised HIF-1α.

### 2.4. Targeting HIFs Impacts on MG63 Differentiation

**Figure 5:**
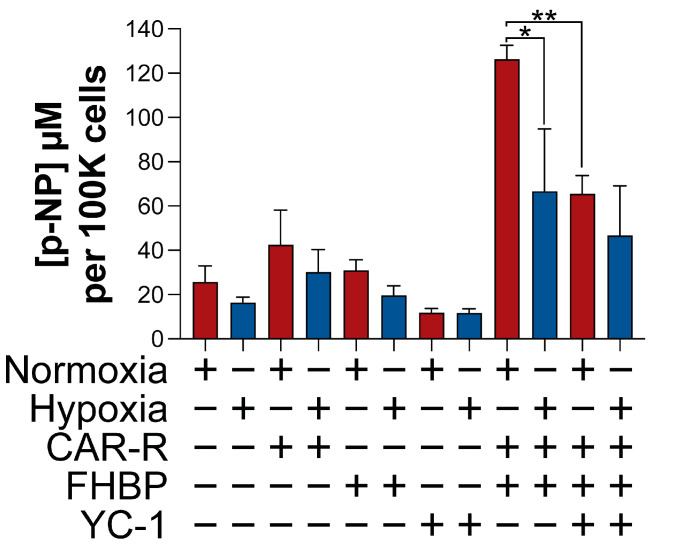
The HIF inhibitor, YC-1, attenuates MG63 differentiation. Hypoxia, which inhibited OS cell differentiation (Figure 3B), is associated with enhanced expression of HIFs (Figure 4). To ascertain if HIFs might be responsible, MG63s were treated with YC-1 (25 μM), a HIF-1α inhibitor. Cells were treated with either CAR-R (100 nM), FHBP (500 nM), or their combination under normoxic or hypoxic conditions for 72 h. Hypoxia significantly reduced the extent of MG63 differentiation (* *p*< 0.01) as supported by a lower concentration of p-nitrophenol (p-NP). Of additional note is the finding that YC-1 significantly inhibited the differentiation of co-stimulated MG63 cells under conventional, normoxic, conditions (** *p* < 0.0001). All data are the mean and standard deviation from two, pooled, independent experiments (N = 8).

**Figure 6:**
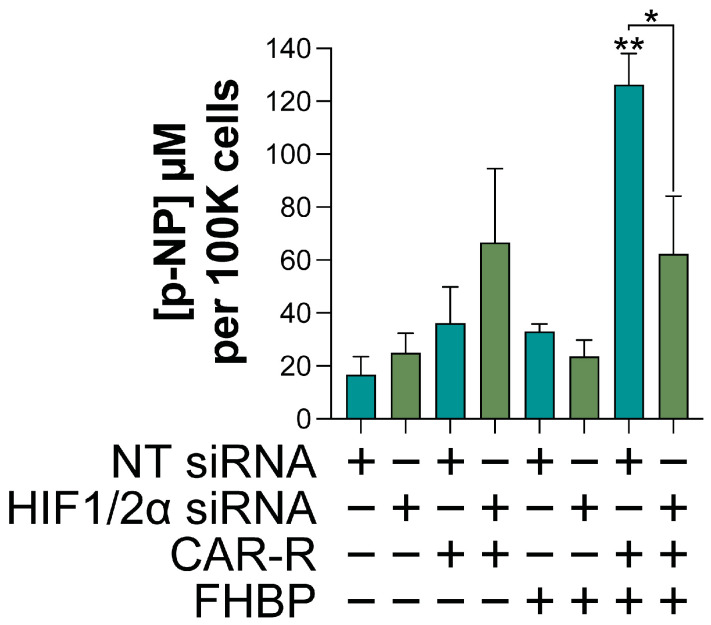
Silencing HIFs compromises MG63 differentiation. In light of the findings for the HIF inhibitor, YC-1 (Figure 5), MG63 cells were either transfected with non-targeting (NT) control siRNA or siRNA targeting both HIF-1 and 2α. Cells were subsequently treated, under conventional conditions, with either CAR-R (100 nM), FHBP (500 nM), or their combination. After 72 h, cells were processed to ascertain the extent of differentiation via a total alkaline phosphatase (ALP) assay. The increased concentration of p-nitrophenol (p-NP) reflects greater ALP activity and, therefore, cellular maturation. Co-stimulated cells transfected with NT siRNA displayed significantly more p-NP compared to all other non-targeted groups (** *p* < 0.001). Conversely, co-treated cells transfected with targeting siRNA had significantly less p-NP compared to the non-targeting counterpart (* *p* < 0.01). All data are the mean and standard deviation from two, pooled, independent experiments (n = 12).

Hypoxia, which inhibited MG63 differentiation (Figure 3), is associated with an enhanced expression of HIFs (Figure 4). To ascertain if HIFs might be responsible, cells were exposed to 3-(5’-hydroxymethyl-2’-furyl)-1-benzylindazole (YC-1, 25 μM), an inhibitor of HIFs. The application of YC-1 did not prevent the significant reduction (*p* < 0.01) in ALP activity for CAR-R-FHBP co-treated, hypoxic cells (Figure 5). Of further significance was the finding that YC-1 reduced the extent of MG63 differentiation under normoxic conditions. A complimentary study utilising siRNA targeting both HIF-1 and 2α led to a similar outcome; siRNA non-targeting controls underwent differentiation in response to CAR-R-FHBP co-treatment, whereas the siRNA targeting counterpart displayed significantly less (*p* < 0.01) ALP activity (Figure 6).

### 2.5. MG63 VDR Expression in Response to Hypoxia and VDR Agonists

**Figure 7:**
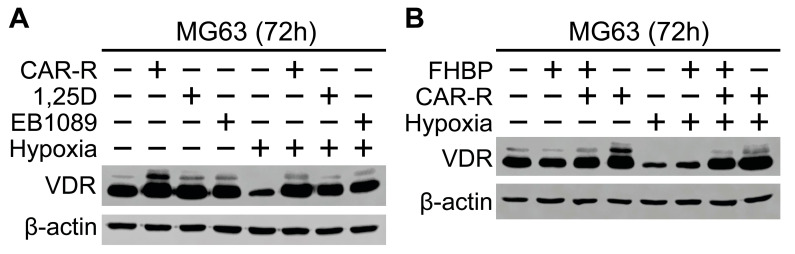
Expression of MG63 VDR—influence of hypoxia and VDR agonists. (**A**). MG63 cells, seeded into T25 flasks, were treated with either CAR-R, 1,25D, or EB1089 (100 nM) under normoxic and hypoxic conditions for 72 h. Flasks were subsequently removed, the media aspirated, and the cell monolayers processed for SDS-PAGE and Western blotting. Blots were probed for the VDR alongside β-actin, which served to confirm equal sample loading. Compared to the unstimulated, normoxic control, VDR expression is markedly less for hypoxic cells. However, treatment of hypoxic cells with each of the VDR agonists substantially increases VDR levels. (**B**). Cells were maintained under normoxic and hypoxic conditions and treated with either CAR-R (100 nM), FHBP (500 nM), or their combination for 72 h. As per A, the hypoxic unstimulated control had much lower VDR levels compared to the normoxic control. FHBP alone appears to be without effect on VDR expression. Both blots are a representative from four experiments.

As expected, MG63 cells exhibited clear evidence of VDR protein expression (Figure 7). Exposure of the cells to a hypoxic environment led to a marked reduction in VDR levels. However, when MG63 cells were treated with either CAR-R, 1,25D or EB1089, the cells responded by increasing VDR protein production, comparable to that observed for normoxic cells.

## 3. Discussion

Osteosarcoma (OS), a mesenchymal malignant bone tumour, primarily affecting adolescents, continues to have a bleak prognosis compared to other cancers. Indeed, survival improvement has not been forthcoming for decades, despite the enormous amount of time and energy invested into randomised clinical trials [14]. A major factor responsible for poor OS patient survival is resistance to chemotherapy [15], a phenomenon compounded by hypoxia [16]. The general consensus, therefore, is to identify novel approaches towards improving treatment outcomes for OS.

In taking these steps, we examined the antiproliferative and pro-differentiating potential of CAR-R, a non-calcaemic, potent agonist of the VDR [7,17]. We used MG63 cells, an OS cell line sourced from a juxtacortical lesion of the left femur from a 14-year-old male [18]. Our focus on CAR-R has been informed from the expanding literature reporting on the promising anti-cancer properties of vitamin D and vitamin D surrogates [3,5,19]. It is widely recognised that 1,25D is antiproliferative; the up-regulation of p21 and p27 and resultant suppression of cyclin D and E in response to 1,25D culminates in G1/G0 cell cycle arrest ([1] and references therein). It has been known for approximately thirty years that LCA, a weak VDR agonist, can cause G1/G0 cell cycle arrest in the human promyelocytic leukaemia cel line, HL60 [20]. The application of a more potent VDR agonist, LCA acetate, inhibited THP-1 cell proliferation and induced their differentiation [21]. Similarly, LCA hydroxyamide was reported to induce cell cycle arrest, at submicromolar concentrations, in cyclin D1-dependent cell types [22]. In a recent study, we found that nanomolar concentrations of a cyanoamide derivative of LCA could inhibit MG63 cell growth comparable to equimolar 1,25D [23]. Collectively, these studies show promise for the use of LCA derivatives in the arsenal against cancer.

Herein, we initially explored if CAR-R could reduce the extent of MG63 growth. Under conventional culturing conditions, the exposure of CAR-R (100 nM) to MG63 cells resulted in significantly fewer cells following a three-day culture. The cell number was reduced by approximately 40%. In parallel, we sought to determine if CAR-R could attenuate the pro-mitogenic effects of FHBP, a stable analogue of the pleiotropic lipid growth factor, LPA. In keeping with our previous studies for VDR agonists [24], the application of CAR-R was able to modestly reduce (~15%) the extent of cell proliferation to FHBP.

Having found that CAR-R could dampen MG63 growth under conventional, normoxic, conditions, we next examined what might happen for hypoxic cells. Solid tumours, including OS, experience hypoxia [11]. The multifaceted nature of hypoxia upon cancer cell survival impacts on chemosensitivity, not only to conventional therapies, but also to 1,25D. There is an expanding body of evidence demonstrating that hypoxia can compromise the beneficial, anti-cancer effects, of 1,25D [13]. Herein, we find that the ability of CAR-R to inhibit MG63 growth was comparable for both normoxic and hypoxic cells, encouraging findings in support of CAR-R as a potential adjunct for OS. Whilst we did not address cell cycle status in this study, it is highly likely that CAR-R reduces the extent of MG63 proliferation via G1/G0 cell cycle arrest akin to the mode of action of 1,25D, LCA and its derivatives.

In a next set of studies, we assessed the potential of CAR-R-FHBP co-treatment to support MG63 differentiation; in isolation, VDR agonists do not fully support MG63 differentiation, they need to be in receipt of an additional stimulus, for example LPA [25], an LPA1/3 receptor agonist [24], or transforming growth factor beta [26]. An evaluation of differentiation was examined under three different settings: normoxia, normoxia with DMOG, and hypoxia. DMOG is a widely used hypoxia mimetic, closely matching the transcriptional responses induced by hypoxia [27]. The compound is often used in parallel with hypoxia station studies in determining the impact of hypoxia/hypoxia-like conditions on cell fate. As we have previously reported for 1,25D, LCA, and LCA derivatives [23,28], the co-stimulation of normoxic MG63s with CAR-R and FHBP resulted in their differentiation, as supported by clear increases in alkaline phosphatase (ALP) activity. In marked contrast, the inclusion of either DMOG or treatment of cells under hypoxic conditions led to a significant reduction in ALP activity. Whilst there was not a complete inhibition of enzyme activity for both models, the findings suggest that hypoxia compromises the ability of CAR-R to fully support OS cell differentiation.

A compelling hallmark of hypoxia is the stabilisation and enhanced activity of hypoxia-inducible factors (HIFs), three transcription factors which serve to support cancer cell survival [16]. Unsurprisingly, we found that hypoxic MG63 cells expressed greater levels of HIF-1α and HIF-2α compared to cells cultured under normoxic conditions. Since hypoxia attenuated the differentiation of MG63 cells, we explored if neutralising HIF activity might restore the differentiation response. To this end, we started with the small molecule YC-1, which inhibits HIF-1α activity in vitro [29]. Of further significance are the findings that YC-1 can reduce tumour size in a variety of murine tumour xenograft models [29]. With regard to OS, YC-1 has been assessed for its ability to enhance MG63 demise to both approved and potential anticancer agents [30,31,32]. Interestingly, the application of YC-1 inhibited MG63 differentiation for both normoxic and hypoxic conditions. Aware of potential off-target effects brought about by many low-molecular-weight inhibitors, we pivoted to siRNA knockdown experiments. As with YC-1, SiRNAs targeting both HIF-1 and 2-α resulted in a compromised differentiation response, whereas non-targeting, control siRNA was without effect.

For normoxic cells, we found evidence that FHBP stimulated an increase in HIF-1α, the level increasing further for FHBP-CAR-R co-treated cells. A modest increase was also observed for HIF-2α in this group. There are reports that LPA can stabilise and promote the expression of HIF-1α in a variety of in vitro models (e.g., [33,34,35,36,37,38,39]). In light of these studies and for the findings presented herein, it is tempting to speculate that HIFs might be important for MG63 differentiation, albeit in response to FHBP-CAR-R co-treatment.

In a final set of studies, we ascertained the expression of the VDR protein via Western blotting. Hypoxia led to a clear reduction in the VDR protein expression for unstimulated cells. However, the enhanced expression of the VDR was clearly evident for hypoxic MG63 cells exposed to either 1,25D, the VDR agonist EB1089 [5], or CAR-R. It is well known that 1,25D enhances the expression of the VDR through a process referred to as autoregulation [40,41,42,43]. Over thirty years ago, it was initially reported that 1,25D could stimulate expression of the VDR protein in MG63 cells [44]. We now report on VDR protein expression in response to VDR agonists in a hypoxic setting. The finding that hypoxic MG63 cells are able to up-regulate VDR protein expression in response to CAR-R is encouraging. Of additional significance are the findings that CAR-R can inhibit hypoxic MG63 growth. However, whilst CAR-R was found to bolster VDR expression under hypoxic conditions, the observation for an attenuated differentiation response is perplexing. Further studies will be required to see if the differentiation response under hypoxia can be restored to a level typically observed for normoxic cells. In doing so, there is a real possibility that less calcaemic VDR agonists could be included in the management of OS. Realising this will necessitate future studies using other OS cell lines, e.g., Saos-2 and 143B, alongside primary human osteoblasts.

## 4. Materials and Methods

### 4.1. General

The methodological details provided for Section 4.1, Section 4.2, Section 4.7 and Section 4.8 are essentially similar to that reported by us previously [23]. These details have been preserved here for the sake of clarity and experimental reproducibility. Unless stated otherwise, all reagents were of analytical grade from Sigma–Aldrich (Gillingham, Dorset, UK). Stocks of (3S)1-fluoro-3-hydroxy-4-(oleoyloxy)butyl-1-phosphonate (FHBP, catalogue number L-9118, Tebu-bio, Peterborough, UK), a phosphatase-resistant lysophosphatidic acid (LPA) analogue, were prepared in 1:1 ethanol/tissue culture grade water to a final concentration of 500 μM and stored at −20 °C. Dimethyloxalylglycine (DMOG, Cambridge Bioscience, Cambridge, UK) was reconstituted to 10 mM in serum-free tissue culture media, aliquoted, and stored at −20 °C. Stocks of 1,25D and EB1089 (100 μM) were prepared in ethanol and stored at −20 °C. CAR-R was synthesised from LCA as detailed previously [7] and stocks (100 μM) prepared in DMSO and likewise stored at −20 °C. For all experiments, CAR-R and DMOG were used at a final concentration of 100 nM and 250 μM, respectively, as informed from initial pilot studies.

### 4.2. Human Osteosarcoma (OS) Cells

Human OS cells (MG63, ECACC, Porton Down, Salisbury, UK) were cultured in conventional tissue culture flasks (250 mL, Greiner, Frickenhausen, Germany) in a humidified atmosphere at 37 °C and 5% CO_2_. Cells were grown to confluence in Dulbecco’s modified Eagle medium (DMEM)/F12 nutrient mix (Gibco, Paisley, UK, catalogue number 21331-020) supplemented with sodium pyruvate (1 mM final concentration), L-glutamine (4 mM), streptomycin (100 ng/mL), penicillin (0.1 units/mL) and 10% *v*/*v* foetal calf serum (Gibco, Paisley, UK). The growth media (500 mL final volume) was also supplemented with 5 mL of a 100× stock of non-essential amino acids. Once confluent, MG63s were subsequently dispensed into blank 24-well plates (Greiner, Frickenhausen, Germany). In each case, wells were seeded with 1 mL of 10 kcells/mL suspension (as assessed by haemocytometry). Cells were then cultured for 3 days, and the media was then removed and replaced with serum-free, phenol red-free DMEM/F12 (SFCM, Gibco, Paisley, UK, catalogue number 11039-021) to starve the cells overnight. MG63s were subsequently treated with CAR-R (100 nM), FHBP (500 nM), or a combination of these factors in SFCM supplemented with 500 μg/mL fatty acid-free human serum albumin. The rationale for CAR-R-FHBP co-treatment is to promote cellular differentiation, as we have previously conducted for related works [23,24,25]. The medium used for all the different treatments was SFCM to eliminate any interference with the assays described below. After the desired time point (72 h), the monolayers were processed to assess the cell number and total ALP activity to ascertain the extent of cellular maturation. Responses of cells to both normoxic and hypoxic conditions were conducted.

For the Western blotting studies, cells were seeded into T25 culture flasks, with 7 mL of a 10 kcells/mL suspension per flask. After a 3-day culture, the medium was removed and replaced with SFCM to starve the cells overnight. Once starved, the cells were treated and flasks either left under conventional, normoxic conditions or transferred to a hypoxia station.

### 4.3. Hypoxia Treatments

Treatments of MG63 cells in hypoxia (1% O_2_) were performed in a Don Whitley H45 Hypoxystation using an atmosphere containing 94% N_2_, 5% CO_2_, and 1% O_2_ at 37 °C. A humidity of approximately 75% was maintained during the culture period.

### 4.4. Gene Knockdown Studies Using siRNA

Small interfering RNA (siRNA) transfections were performed using Lipofectamine RNAiMax in serum-free Opti-MEM (Fisher Scientific, Paisley, UK). Prior to transfection, MG63 cells were placed in penicillin-streptomycin-free media for 24 h. Cells were then transfected with siRNA (final concentration 20 nM) overnight; normal growth medium was added the following day. Cells were left for 48 h to allow gene silencing before the treatment with normoxia or hypoxia. The following siRNAs were used: Negative control siRNA (ON-TARGETplus Non-targeting siRNA pool D-001810-10 Dharmacon and/or Negative control RNA Duplex, 1027310, Qiagen, Manchester, UK), *HIF1A* siRNA (SMARTpool: ON-TARGETplus HIF1A siRNA J-004018-08, Dharmacon), or *EPAS1* (encoding HIF-2a) siRNA (FlexiTube siRNA EPAS1_2 S100380212 Qiagen, Manchester, UK).

### 4.5. Protein Extraction, Quantification, and Sample Preparation

Cells were washed with ice-cold PBS and total cellular protein was extracted from MG63 cells using Cell Signalling Technology (CST) lysis buffer (#9803) supplemented with protease inhibitors (Roche cOmplete mini, Merck, Gillingham, Dorset, UK). Lysates were kept on ice before being stored at −80 °C. Protein quantification was performed using the Bio-Rad DC protein assay kit (Bio-Rad Laboratories Ltd, Watford, Hertfordshire, UK) to achieve an equal protein concentration between samples.

### 4.6. Western Blotting

A Western blot analysis was performed as described previously [45]. Briefly, 50 µg of protein per sample was resolved using sodium dodecyl sulphate–polyacrylamide gel electrophoresis (SDS–PAGE) and transferred to an Immobilon-P polyvinylidene difluoride membrane (Fisher Scientific, Paisley, UK). The antibodies used were as follows: HIF-1a (1:1000, BD, 610,959), HIF-2a (1:1000, CST, 7096), b-actin (1:10,000, Sigma, A5316), and VDR (1:1000, CST, 12,550). Following primary and HRP-conjugated secondary antibody incubation, membranes were incubated with chemiluminescent HRP substrate (Immobilon) and imaged on an LI-COR Odyssey XF Imaging system.

### 4.7. Cell Number

This involved treating the cells with a combination of the tetrazolium compound 3-(4,5-dimethylthiazol-2-yl)-5-(3-carboxymethoxy-phenyl)-2-(4-sulfophenyl)-2H-tetrazolium, inner salt (MTS, Promega, Southampton, UK) and the electron-coupling reagent phenazine methosulphate (PMS). Each compound was prepared separately in pre-warmed (37 °C) phenol red-free, serum-free DMEM/F12, allowed to dissolve, and then combined so that 1 mL of a 1 mg/mL solution of PMS was combined to 19 mL of a 2 mg/mL solution of MTS. A stock suspension of MG63s (1 × 10^6^ cells/mL) was serially diluted in SFCM to give a series of known cell concentrations down to 25 × 10^3^ cells/mL. Each sample (0.5 mL in a microcentrifuge tube) was spiked with 0.1 mL of the MTS/PMS reagent mixture and left for 45 min within a tissue culture cabinet. Once incubated, the samples were centrifuged at 900 rpm to pellet the cells and 0.1 mL of the supernatants were dispensed onto a 96-well microtitre plate and the absorbances read at 492 nm using a multiplate reader. Plotting the absorbances against the known cell number, as assessed initially using haemocytometry, enabled the extrapolation of cell numbers for the experiments described herein.

### 4.8. ALP Activity—A Marker of MG63 Differentiation

As osteoblastic cells move towards a more mature, or differentiated phenotype, they express greater levels of ALP [46,47], an enzyme essential for the provision of a mechanically robust, mineralised bone matrix [48]. An assessment of ALP activity is reliably measured by the generation of p-nitrophenol (p-NP) from p-nitrophenylphosphate (p-NPP) under alkaline conditions. Briefly, the MTS/PMS reagent was removed and the monolayers incubated for a further 5 min in fresh phenol red-free, serum-free DMEM/F12 (0.2 mL/well); this was repeated a second time to remove the residual formazan. Following this incubation period, the medium was removed and the monolayers treated with 0.1 mL/well of 7 mM sodium carbonate, 3 mM sodium bicarbonate (pH 10.3) supplemented with 0.1% (*v*/*v*) Triton X-100 to lyse the cells. After 2 min, each well was treated with 0.2 mL of 15 mM p-NPP (di-Tris salt, Sigma, UK) in 70 mM sodium carbonate, 30 mM sodium bicarbonate (pH 10.3), supplemented with 1 mM MgCl_2_. Lysates were then left under conventional cell culturing conditions for 1 h. After the incubation period, 0.1 mL aliquots were transferred to 96-well microtitre plates, and the absorbance was read at 405 nm. An ascending series of p-NP (50–400 μM) prepared in the incubation buffer enabled the quantification of product formation.

### 4.9. Statistical Analysis

Unless stated otherwise, all experiments described above were performed at least three times, on different days using different passage numbers of cells. For Figure 2, Figure 3, Figure 5 and Figure 6, the minimum replicate number, per treatment group, was four. When comparing two groups, the statistical significance was determined by an unpaired, two-tailed Student’s *t*-test, assuming unequal variance. Data presented in Figure 2A were subject to a one-way analysis of variance (ANOVA). Data are presented as the mean and the standard deviation. Data were deemed to be statistically significant when *p* < 0.05. GraphPad Prism 10.4 was used for data analysis.

## Data Availability

The datasets generated for this study are available from J.P.M. on reasonable request.
